# Potassium Channel Activator Attenuates Salicylate-Induced Cochlear Hearing Loss Potentially Ameliorating Tinnitus

**DOI:** 10.3389/fneur.2015.00077

**Published:** 2015-04-07

**Authors:** Wei Sun, Jun Liu, Chao Zhang, Na Zhou, Senthilvelan Manohar, Wendy Winchester, Jason A. Miranda, Richard J. Salvi

**Affiliations:** ^1^Center for Hearing and Deafness, State University of New York at Buffalo, Buffalo, NY, USA; ^2^Department of Otolaryngology, General Hospital of PLA, Beijing, China; ^3^Department of Otolaryngology, Peking University Third Hospital, Beijing, China; ^4^Neusentis Research Unit, Pfizer, Cambridge, UK

**Keywords:** salicylate, KCNQ, hearing loss, compound action potential, otoacoustic emission

## Abstract

High dose sodium salicylate causes moderate, reversible hearing loss and tinnitus. Salicylate-induced hearing loss is believed to arise from a reduction in the electromotile response of outer hair cells (OHCs) and/or reduction of KCNQ4 potassium currents in OHCs, which decreases the driving force for the transduction current. Therefore, enhancing OHC potassium currents could potentially prevent salicylate-induced temporary hearing loss. In this study, we tested whether opening voltage-gated potassium channels using ICA-105665, a novel small molecule that opens KCNQ2/3 and KCNQ3/5 channels, can reduce salicylate-induced hearing loss. We found that systemic application of ICA-105665 at 10 mg/kg prevented the salicylate-induced amplitude reduction and threshold shift in the compound action potentials recorded at the round window of the cochlea. ICA-105665 also prevented the salicylate-induced reduction of distortion-product otoacoustic emission. These results suggest that ICA-105665 partially compensates for salicylate-induced cochlear hearing loss by enhancing KCNQ2/3 and KCNQ3/5 potassium currents and the motility of OHCs.

## Introduction

Aspirin, with sodium salicylate as an active ingredient, is one of the most widely used analgesic, anti-inflammatory, and antipyretic drugs (1). Sodium salicylate at a high dose (1–10 mM) can induce a moderate, reversible cochlear hearing loss (~20–40 dB threshold elevation), tinnitus, and suppression of otoacoustic emissions (OAEs) (2, 3). These perceptual changes have generally been attributed to functional impairments in the cochlea such as a down regulation of the electromotile response of outer hair cells (OHCs) and a decrease in neural output of the cochlea (4–9). The effect of salicylate on OHC electromotility at a high concentration (>1 mM) is likely due to its competition for the chloride anion binding site on the prestin motor protein, rather than plasma membrane mechanics (7). A more recent study found that at moderate concentrations (0.1–1 mM), salicylate also causes a concentration-dependent reversible reduction of a voltage-gated potassium channel, which is encoded by KCNQ4 gene of OHCs (10). This subsequently may depolarize the OHC, which would reduce the net driving force of the transduction current thereby diminishing OHC electromotility. Thus, there are at least two mechanisms by which salicylate can cause an OHC-based cochlear hearing loss.

KCNQ genes encode a family of five voltage-gated potassium channels, i.e., KCNQ1 through KCNQ5, and KCNQ channel has six transmembrane domains and a single pore loop (11). In the cochlea, all members of the KCNQ family have been identified (11, 12). KCNQ4 channels are expressed on hair cells and spiral ganglion neurons (13, 14) and the mutation of KCNQ4 accounts for human non-syndromic deafness DFNA2 an autosomal dominant hearing loss (15). Since the resting potential of OHC is dependent on potassium currents, KCNQ4 mutation can reduce *I*_K_, which leads to selective degeneration of OHCs (16). In addition, KCNQ2 is expressed in the modiolus and organ of corti, synaptic regions under hair cells, spiral ganglion auditory neurons, and nerve fibers innervating hair cells (12). KCNQ3 was detected in the cochlear lateral wall. Although the immunocytochemistry study did not clearly identify KCNQ2/3 on OHCs, M-like currents, which are mediated by KCNQ2/3 and KCNQ3/5 channels (17–20), have been recorded in isolated guinea pig OHCs (21). M-currents are low-threshold depolarization-activated potassium currents that can be inhibited by the cholinergic agonist muscarine (22). M-currents play a role in stabilizing the resting membrane potential, which is important for regulating cellular excitability and K+ homeostasis (23). However, the function of KCNQ2/3 and KCNQ3/5 in salicylate-induced cochlear hearing loss is poorly understood. The recently identified KCNQ5 channels, often co-expressed with KCNQ3 channels, are expressed in the synaptic endings in the auditory brainstem and the calyx terminals of type I vestibular hair cells (24). Therefore, we tested the effects of ICA-105665, a novel small molecule that selectively opens KCNQ2/3 and KCNQ3/5 channels (Kv7.2/7.3 and Kv7.3/7.5) (25, 26) to determine its effects on salicylate-induced hearing loss.

## Experimental Procedures

### Animals

Twenty-one adult male Sprague-Dawley rats (3–5 months, 200–400 g) were used for the physiological recordings. All protocols were approved by the Institutional Animal Care and Use Committee (IACUC) of the State University of New York at Buffalo that conform to the guidelines issued by the National Institutes of Health. This research minimized the number of animals used and their suffering.

### Surgery

Rats were anesthetized with a mixture of ketamine (50 mg/kg) and xylazine (6 mg/kg). The bulla of the left ear was exposed and a silver ball electrode was placed on the round window (27). Body temperature was measured and maintained using a heating pad (Homeothermic blanket control unit, Harvard Apparatus, MA, USA). Compound action potentials (CAPs) were recorded before and after the delivery of sodium salicylate (250 mg/kg, i.p.) with/without ICA-105665 (10 mg/kg, i.m.). Since the peak concentration of ICA-105665 occurred ~1 h after the injection (manuscript in preparation), ICA-105665 was always injected 1 h before salicylate treatment. Sodium salicylate (S-2679, Sigma, St. Louis, MO, USA) was dissolved in Ringer’s solution (25 mg/ml). ICA-105665 (100 mg) was first dissolved in 50 μl of DMSO and 950 μl of methylcellulose, and then dissolved in 19 ml of water to make a final ICA-105665 concentration of 5 mg/ml. This dose resulted in a mean ICA-105665 unbound plasma concentration of 69.1 nM ± 9.5 SEM, which is a pharmacologically relevant level for selectivity of this compound. The ICA-105665 *in vitro* EC50 (concentration at which 50% of the maximum effect is achieved) for cloned rat KCNQ2/3 channels is 160 nM with high selectivity at this exposure level (25) (manuscript in preparation).

### Acoustical stimuli and recordings

Stimuli were generated with hardware from Tucker-Davis Technologies (TDT, Alachua, FL, USA) and software (SigGen, TDT) and presented through a high frequency speaker (Fostex FT28D, Fostex, Tokyo, Japan). Tone-bursts (2 ms duration and 1 ms rise/fall) at 4, 8, 12, 16, and 32 kHz were used to elicit the CAP at intensities ranging from 25 to 95 dB SPL (10 dB step). Stimuli were calibrated using a sound level meter (824, Larson Davis, Depew, NY, USA) with a half inch microphone (Larson Davis, model #2559).

The output of the silver ball electrode used to record the CAP was connected to a preamplifier (RA16LA, TDT) using a flexible, low noise cable. The output of the preamplifier was delivered to a digital signal processing module (RX5-2, Pentusa Base Station, TDT) connected to a computer. The electrical response was digitized (25 kHz), filtered (100–3000 Hz), and averaged (*n* = 1024) over a 10 ms sampling window.

### DPOAE recordings

The distortion-product otoacoustic emissions (DPOAE) were recorded in a separate group of rats (*n* = 8) using a Smart DPOAE system (Intelligent Hearing Systems, FL, USA). A pair of continuous tones (F1 and F2) was used to elicit the DPOAE. The F2 testing frequencies were from 4 to 24 kHz and the ratio of F2 to F1 was set at 1.2. The F2 testing intensities ranged from 15 to 60 dB with 5 dB step (the intensity of F1 was 10 dB higher than F2). In four rats, the DPOAEs were recorded prior to and 1 h after salicylate treatment. In the other four rats, the baseline DPOAEs was recorded and then the rats were treated with ICA-105665 (10 mg/kg, i.m.). One hour after ICA-105665 treatment, DPOAEs were recorded and then the rats were treated with salicylate (250 mg/kg, i.p.) and DPOAEs were recorded 1 h afterward.

### Statistic data analysis

The graphs and statistical analyses were performed using GraphPad Prism version 5.00 for Windows (GraphPad Software, San Diego, CA, USA) unless otherwise noted. Results are presented as mean ± SEM.

## Results

### ICA-105665 attenuated salicylate’s effect on cochlea

**Figure 1:**
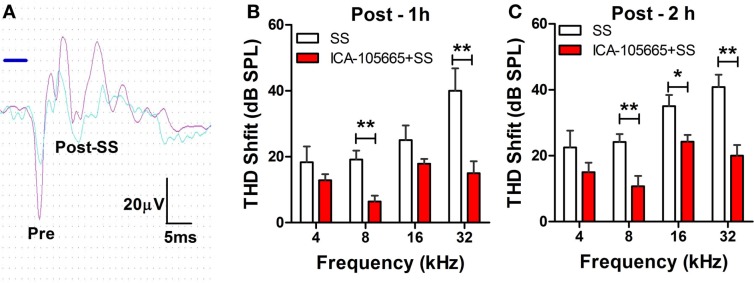
**Effects of ICA-105665 on compound action potential (CAP) threshold shifts induced by salicylate (SS)**. **(A)** Typical CAP response recorded from the round window of cochlea elicited by a tone-burst stimulus (8 kHz, 80 dB SPL). **(B,C)** Significant threshold shifts of CAP were induced by high doses of SS (250 mg/kg, i.p.). ICA-105665 (10 mg/kg, i.p., *n* = 7) treatment caused a significant reduction of CAP threshold shifts at 8, and 32 kHz on 1 h, and 8, 16, and 32 kHz on 2 h after salicylate injection (Student’s *t*-test, df = 11, *P* values were listed in Table 1).

The threshold and the amplitude of CAPs recorded from the round window of anesthetized rats were monitored before, and at 1 and 2 h after treatment with salicylate (250 mg/kg, i.p.) with/without ICA-105665 (10 mg/kg, i.p.). Salicylate treatment caused a large reduction of the CAP amplitude and noticeable threshold shifts. Figure 1A shows typical CAP waveforms recorded before and after salicylate treatment. Figures 1B,C show the threshold shifts of the CAP at 1 and 2 h after salicylate injection. The CAP threshold significantly increased 1–2 h after salicylate injection. The average CAP threshold shifts were 18 ± 4.7, 19 ± 2.7, 25 ± 4.4, and 40 ± 6.8 dB (*n* = 6) at 4, 8, 16, and 32 kHz 1 h after salicylate injection and slightly less at 2 h. To determine if ICA-105665 could prevent salicylate-induced hearing loss, a separate group of rats were treated with ICA-105665 (10 mg/kg, i.p., *n* = 7) 1 h before 250 mg/kg salicylate administration. One hour after ICA-105665 was injected, the CAP threshold showed no obvious shift (the average CAP shift was 0.8 ± 0.8, 6.6 ± 4.9, 6.6 ± 3.3, and 2.8 ± 3.2 dB at 4, 8, 16, and 32 kHz, respectively, *n* = 6). The CAP threshold and amplitude were measured 1–2 h after salicylate injection. The CAP threshold shifts were 14 ± 1.7, 6.4 ± 1.5, 17 ± 1.7, and 15 ± 3.6 dB at 4, 8, 16, and 32 kHz 1 h after ICA-105666 plus salicylate treatment. There was a significant difference between the salicylate treated group and the ICA-105665 plus salicylate treated group at 8 and 32 kHz on 1 h and 8, 16, and 32 kHz on 2 h after salicylate injection (Student’s *t*-test, df = 11, *P* values were listed in Table 1). There was no significant difference at 4 kHz on 1 and 2 h, and 16 kHz on 1 h treatment (Table 1).

**Table 1:** **Student’s *t*-test on CAP threshold shifts caused by salicylate (250 mg/kg, i.p.) vs. salicylate plus ICA-105665 (df = 11)**.

	4 kHz	8 kHz	16 kHz	32 kHz
1 h treatment	*P* = 0.14	*P* = 0.001**	*P* = 0.06	*P* = 0.006**
2 h treatment	*P* = 0.11	*P* = 0.007**	*P* = 0.017*	*P* = 0.001**

*P < 0.05, **P < 0.01.

**Figure 2:**
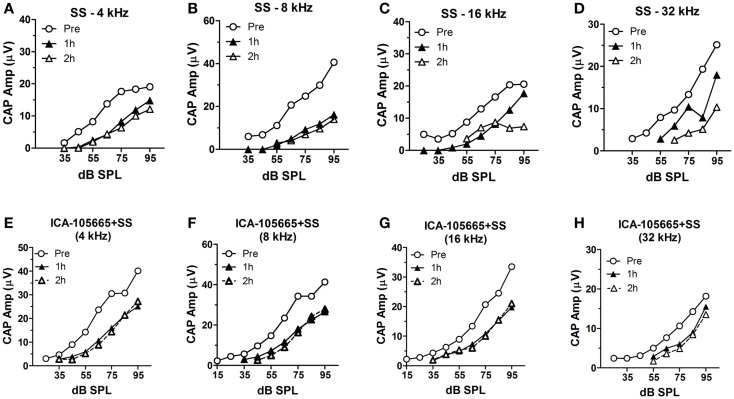
**The input–output functions of CAP before and after salicylate treatment with/without ICA-105665**. **(A–D)** The CAP amplitude change induced by salicylate treatment (250 mg/kg, i.p.) at 4, 8, 16, and 32 kHz. **(E–H)** The CAP amplitude change caused by salicylate and ICA-105665 (10 mg/kg, i.p., *n* = 7) at 4, 8, 16, and 32 kHz.

**Figure 3:**
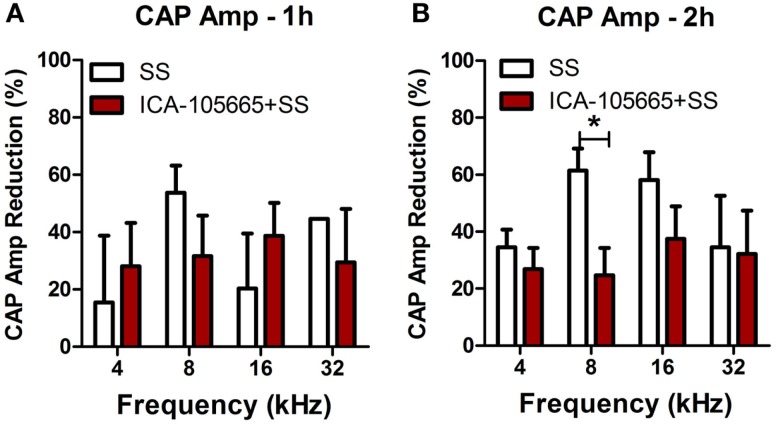
**The reduction of CAP amplitude at 95 dB SPL induced by salicylate with/without ICA-105665 treatment**. There was no significant difference between the CAP amplitude reduction in the salicylate alone group with the salicylate plus ICA-105665 group at 1 h post-treatment (**A**, Student’s *t*-test, *P* > 0.05), but there was a significant difference at 8 kHz for the 2 h treatment groups (**B**, Student’s *t*-test, *t* = 2.9, df = 9).

The CAP amplitudes were measured in both groups before and after salicylate injection (Figures 2A–H). The CAP amplitude dropped significantly at 4, 8, 16, and 32 kHz after salicylate injection with/without ICA-105665 application (10 mg/kg). The average amplitude decrease caused by salicylate with/without ICA-105665 was evaluated at 95 dB SPL. Salicylate caused a mean (±SEM, *N* = 5) decrease of 34 ± 6, 61 ± 7, 58 ± 10, and 33 ± 23% at 4, 8, 16, and 32 kHz 2 h after salicylate treatment (*n* = 5). In the ICA-105665 plus salicylate group, the CAP mean (±SEM, *n* = 5) amplitude decrease at 95 dB SPL was 27 ± 7, 25 ± 9, 34 ± 13, and 36 ± 16% decrease 2 h after salicylate treatment (*n* = 6). Figure 3 shows the mean reduction of CAP amplitude at 95 dB SPL-induced salicylate with/without ICA-105665 treatment. There was no significant difference between the CAP amplitude reduction in the salicylate alone group with the salicylate plus ICA-105665 group at 1 h post-treatment (Student’s *t*-test, *P* > 0.05), but there was a significant difference at 8 kHz for the 2 h treatment groups (Student’s *t*-test, *P* = 0.017, *t* = 2.9, df = 9).

### Distortion-product otoacoustic emission

**Figure 4:**
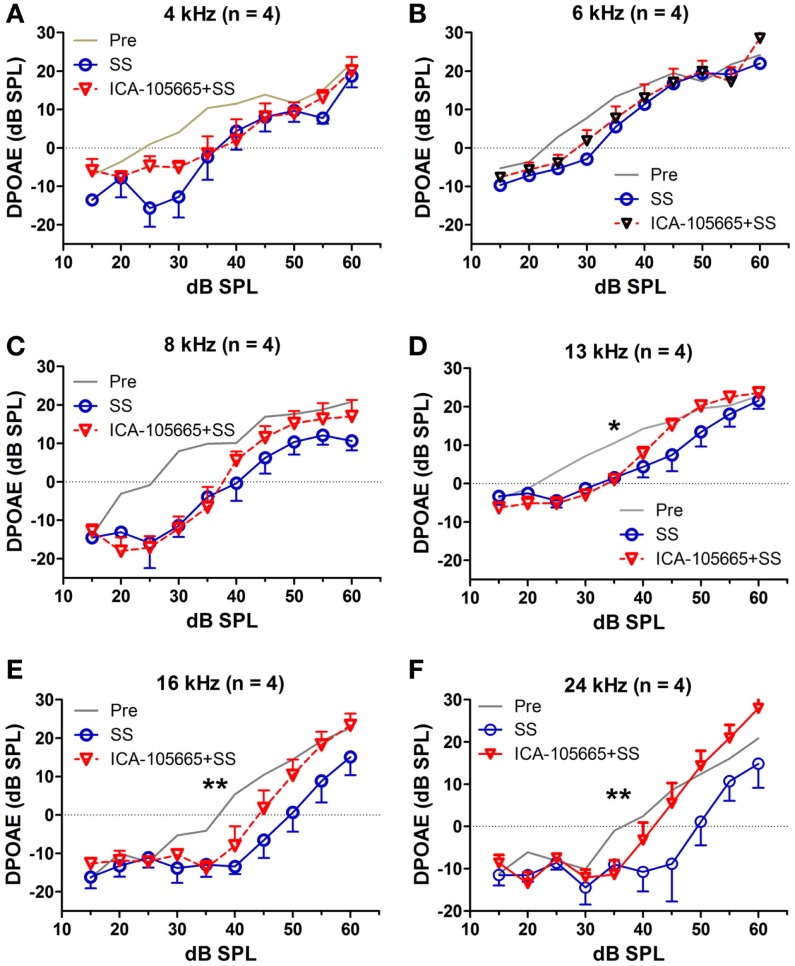
**The effects of salicylate and ICA-105665 on the amplitudes of distortion-product otoacoustic emissions (DPOAE)**. **(A–F)** Salicylate induced a significant decrease in DPOAE amplitude at all frequencies (4–24 kHz, two-way ANOVA, *P* < 0.001). Treatment with ICA-105665 prior to salicylate injection lessened the SS-induced DPOAE reductions at high frequencies. The slopes of the DPOAE amplitude–intensity functions before and after the treatment of salicylate with/without ICA-105665 were significant at 13, 16, and 24 kHz, but not on 4, 6, and 8 kHz (the *F* and *P* values were listed in Table 2).

**Figure 5:**
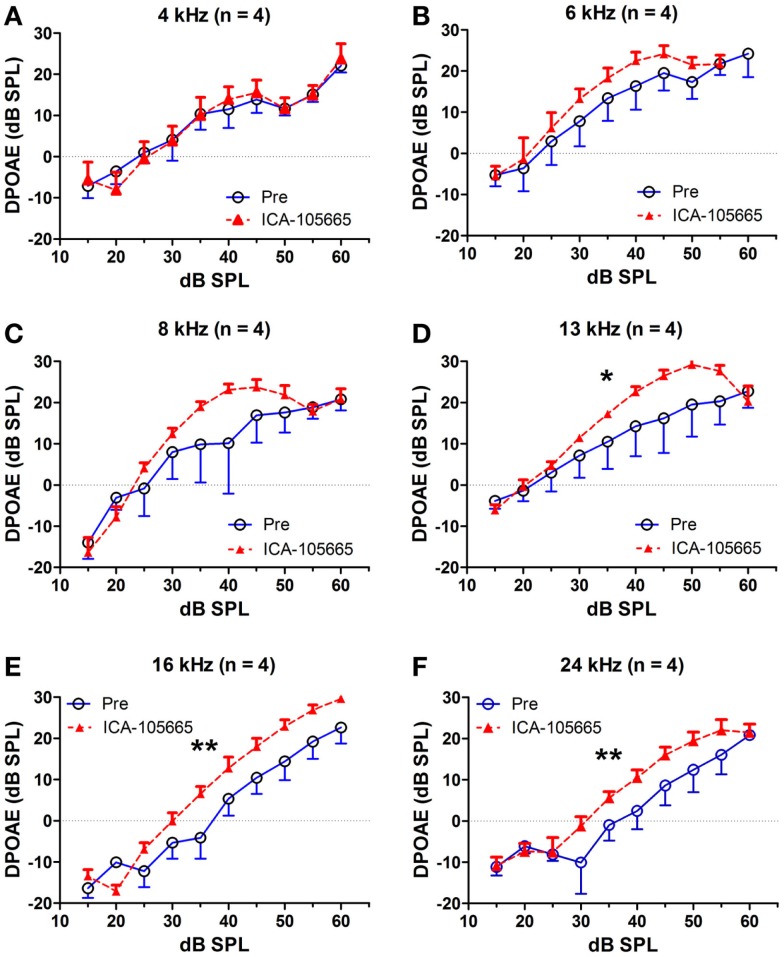
**ICA-105665 treatment showed a slight increase on DPOAE amplitudes at high frequencies (D–F), not at low frequencies (A–C)**. However, the difference was not significant due to the small sample size.

The effects of salicylate, ICA-105665 and ICA-105665 plus salicylate on DPOAE (4–24 kHz), are shown in Figures 4 and 5. Salicylate induced a decrease in DPOAE amplitude at all tested frequencies and more changes were found in the high frequencies. To determine if the ICA-105665 could prevent the salicylate-induced DPOAE decrease, rats were treated with ICA-105665 (10 mg/kg, i.p., *n* = 4) and then with salicylate 1 h later. After ICA-105665 plus salicylate treatment, the DPOAE amplitudes were significantly larger than those in the salicylate alone group at 16 and 24 kHz (40–60 dB SPL). Since the increase in DPOAE amplitude with ICA-105665 plus salicylate vs. salicylate alone mainly occurred at higher intensities, we did linear regression analysis on the DPOAE amplitude–intensity function at 40–60 dB SPL for each animal on each frequency. The slopes of the DPOAE amplitude–intensity functions were used for statistical analysis. We found that the slope of the DPOAE amplitude–intensity function (40–60 dB SPL) before and after the treatment of salicylate with/without ICA-105665 were significantly different at 13, 16, and 24 kHz, but not on 4, 6, and 8 kHz (the *F* and *P* values were listed in Table 2). Figure 5 showed that after ICA-105665 treatment, the DPOAE amplitude showed a slight increase. However, post Tukey’s tests showed a significant difference at 13, 16, and 24 kHz between before and after SS with/without ICA-105665 treatment. This difference was likely caused by the effect of ICA-105665, which enhanced the amplitude of DPOAE. There was no significant difference between before and after ICA-105665 treatment. This may be due to the small sample size has been tested in the experiment.

**Table 2:** **One-way ANOVA test on the slopes of DPOAE amplitude–intensity function (40–60 dB SPL) before and after the treatment of salicylate (SS) with/without ICA-105665**.

	Pre	SS	SS + ICA	ICA	df	*F*	*P*
4 kHz	0.44 ± 0.20	0.56 ± 0.12	0.38 ± 0.08	0.82 ± 0.19	3,15	2.58	0.12
6 kHz	0.35 ± 0.27	0.47 ± 0.12	0.62 ± 0.20	0.28 ± 0.12	3,15	0.89	0.47
8 kHz	0.46 ± 0.50	0.55 ± 0.17	0.55 ± 0.17	−0.2 ± 0.05	3,15	1.49	0.28
13 kHz	0.42 ± 0.25	0.90 ± 0.10	0.77 ± 0.13	−0.07 ± 0.15	3,15	5.81	0.01*
16 kHz	0.86 ± 0.05	1.44 ± 0.21	1.58 ± 0.12	0.84 ± 0.09	3,15	10.58	0.002**
24 kHz	0.88 ± 0.19	1.41 ± 0.12	1.55 ± 0.18	0.56 ± 0.07	3,15	8.04	0.006**

*P < 0.05, **P < 0.01.

## Discussion

Salicylate-induced sensorineural hearing loss is mainly caused by down regulation of the electromotile response of OHCs, which decreases the neural output of cochlea (4–9). In our experiment, we found that the activation of cochlear potassium channels using ICA-105665, an activator of KCNQ2/3 and KCNQ3/5 channels, can decrease salicylate-induced CAP amplitude reduction and threshold shifts. Since ICA-105665 also caused an increase of DPOAE amplitude, our study suggests that ICA-105665 can compensate for salicylate-induced hearing loss presumably by enhancing the electromotility of OHC through activating potassium currents.

KCNQ2, KCNQ3, and KCNQ5 are co-expressed in many regions of the cochlea (11, 28), including OHCs, spiral ganglion neurons, and the lateral wall of the cochlea (12, 15). KCNQ2/3 and KCNQ3/5 are heteromeric channels that may underlie M-currents, which are important in maintaining neuronal excitability (17). M-like currents have been recorded in isolated OHCs in the guinea pig cochlea (21). These currents can be activated at more depolarized potentials than other voltage-gated K+ channels and may be involved in maintaining auditory excitability. We found that activation of KCNQ2/3 and KCNQ3/5 enhanced DPOAE amplitude; these results suggest that activation of M-channel-mediated potassium currents may be involved in electromotility of OHCs. The enhancements of DPOAE amplitude by ICA-105665 were significant at high, but not low frequencies. This suggests that more KCNQ channels are expressed in the basal than the apical turn of cochlea. The expression of KCNQ4 in the IHCs and spiral ganglion neurons decreased from the base to the apex of the cochlea (13). However, there is no report on the gradient of KCNQ2, KCNQ3, or KCNQ5 expression in the cochlea. Since ICA-105665 can activate both KCNQ2/3 and KCNQ3/5 channels, it is unclear whether one channel or the other is responsible for effects on CAP and DPOAE or whether a combined effect is driving these differences.

KCNQ2/3 and KCNQ5 are also broadly expressed in the brain, including hippocampus, caude-putamen, globus pallidus, cortex, thalamus, hypothalamus, and midbrain cortex (18, 29–31). Reduction of KCNQ-mediated currents can increase neuronal excitability to epileptogenic levels and cause convulsion (32). KCNQ2/3 openers, such as ICA-27243, can shift the activation of KCNQ2/3 to a hyperpolarized potential and prevent epilepsy (33). ICA-105665, a new generation of anti-seizure drug targeting KCNQ2/3 channels, was tested for efficacy in several epilepsy models because of its selectivity for potassium channels (25, 26). Unlike other anti-seizure drugs such as retigabine, which also potentiate GABAa receptor response at slightly higher concentrations, ICA-105665 is more selective and does not affect GABAa receptor responses (33). A recent clinical study reported that ICA-105665 at single doses of 100–500 mg reduced photoparoxysmal responses elicited by 2–60 Hz light flash in patients with epilepsy (34). In our preliminary studies, we also found that ICA-105665 can directly affect the firing properties of neurons in the auditory cortex and the inferior colliculus. However, ICA-105665 failed to reduce salicylate-induced hyperexcitability in the auditory cortex, which suggests that ICA-105665 and salicylate may target different receptors and channels in the central auditory system (data not included).

In summary, ICA-105665, an activator of KCNQ2/3 and KCNQ3/5 channels, reduced the toxic effects of salicylate on the amplitude of the CAP and DPOAE. Since ICA-105665 alone enhanced the amplitude of DPOAEs, we speculate that the drug exerts its protective effects against salicylate ototoxicity by targeting the KCNQ2/3 and or KCNQ3/5 channels. ICA-105665 may be a useful compound in protecting against ototoxicity and noise exposure by enhancing the OHC motility.

## Conflict of Interest Statement

The authors declare that the research was conducted in the absence of any commercial or financial relationships that could be construed as a potential conflict of interest.
